# Metabolic Transformation of Gentiopicrin, a Liver Protective Active Ingredient, Based on Intestinal Bacteria

**DOI:** 10.3390/molecules28227575

**Published:** 2023-11-14

**Authors:** Jie Fu, Hang Yu, Qinglan Guo, Yanan Wang, Hui Xu, Jinyue Lu, Jiachun Hu, Yan Wang

**Affiliations:** State Key Laboratory of Bioactive Substance and Function of Natural Medicines, Institute of Materia Medica, Chinese Academy of Medical Sciences and Peking Union Medical College, Beijing 100050, China; fujie@imm.ac.cn (J.F.); yuhang@imm.ac.cn (H.Y.); guonina@imm.ac.cn (Q.G.); wangyanan@imm.ac.cn (Y.W.); xuhui@imm.ac.cn (H.X.); lujinyue@imm.ac.cn (J.L.); hujiachun@imm.ac.cn (J.H.)

**Keywords:** gentiopicrin, intestinal bacteria, identification, metabolic transformation, LC/MS^n^-IT-TOF, NMR

## Abstract

Gentiopicrin, the main component of the famous Chinese patent medicine Long Dan Xie Gan Wan, has the characteristics of fast absorption in vivo and low bioavailability. Intestinal bacteria play an important role in the absorption and pharmacokinetics of oral drugs. In this study, the metabolic transformation of gentiopicrin by intestinal bacteria was examined. High-performance liquid chromatography coupled with ion trap time-of-flight mass spectrometry (LC/MS^n^-IT-TOF) and nuclear magnetic resonance (NMR) were used, and six metabolites were identified, including reduction products (G-M1, G-M2, G-M4, and G-M6), a hydrolytic product (G-M3), and a dehydration product (G-M5) of gentiopicrin aglycone after hydrolysis, reduction, and dehydration reactions were performed by the intestinal flora. This is the first time that chiral metabolites of gentiopicrin (G-M1 and G-M2) were found in this study. In addition, the precursors of glucuronic acid conjugates previously reported in vivo may have come from the intestinal bacterial metabolites G-M1, G-M2, and G-M3. In addition, the metabolic transformation of gentiopicrin in liver microsomes was studied in vitro, and it was found that gentiopicrin did not undergo metabolic transformation under the action of liver microsomes. It is suggested that gentiopicroside may be metabolized in the intestine. This study provides both new insight regarding the investigation of effective substances and an exploration of the pharmacodynamic and toxicological properties of gentiopicrin.

## 1. Instruction

Long Dan Xie Gan Wan, a traditional Chinese patent medicine, is derived from the famous prescription Gendan xiegan decoction collected in “PAIRED DRUGS” (Yifang Jijie) and has the effects of clearing the liver and gall bladder and alleviating dampness and heat. Long Dan Xie Gan Wan can be used to treat dizziness, red eye, tinnitus, deafness, swelling and pain in the ear, side pain, bitter mouth, red urine, and astringent pain during urination. The prescription contains Gentianae Radix et Rhizoma, Bupleuri Radix, Scutellariae Radix, Gardeniae Fructus, Alismatis Rhizoma, Akebiae Caulis, Plantaginis Semen, Angelicae Sinensis Radix, Rehmanniae Radix, and Glycyrrhizae Radix ET Rhizoma Praeparata Cum Melle, of which Gentianae Radix et Rhizoma is the principal drug. In the 2020 edition of the Chinese Pharmacopoeia, gentiopicrin is listed as one of the quality control ingredients of Long Dan Xie Gan Wan [1].

Gentiopicrin, an iridoid glycoside, has a variety of pharmacological properties, such as liver protection, liver damage prevention [2,3], analgesic and anti-inflammatory activities [4], and glycolipid metabolism improvement [5]. The efficacy of this compound is closely related to its pharmacokinetic characteristics in vivo. It has been reported that gentiopicrin is quickly absorbed in vivo and has low bioavailability in mice [6]. Intestinal bacteria play an important role in the efficacy of compounds with low oral bioavailability [7,8]. As early as 1989, researchers found that gentiopicrin could be metabolized by human intestinal bacteria into erythrocentaurin, gentiopicral, 5-hydroxymethylisochroman-1-one, 5-hydroxymethylisochromen-1-one, and *trans*-5,6-dihydro-5-hydroxymethyl-6-methyl-1*H*,3*H*-pyrano [3,4-*c*]pyran-1-one in an anaerobic environment, all of which are coumarins [9]. Furthermore, the metabolites erythrocentaurin and gentiopicral are formed by the hydrolysis of gentiopicrin under the action of intestinal bacteria, and the enzyme that mediates this reaction might be β-glucosidase [10,11,12]. There are also some reports that gentiopicrin can be metabolized into alkaloid compounds, such as gentianine and gentianal, by human intestinal bacteria [13,14]. Obviously, intestinal bacteria play an important role in gentiopicrin metabolism. In addition, in an in vivo metabolic transformation study, 2 uronic acid conjugate metabolites of gentiopicrin were found in rat plasma and 17 metabolites were detected in urine, which formed after hydrolysis, oxidation, *N*-heterocyclylation, or glucoaldehyde aldehyde acidification after the gavage of gentiopicrin [15,16]. Thus, we can infer that gentiopicrin can be widely metabolized in vivo and in vitro. Gentiopicrin metabolites have also been reported to have potential pharmacological activities. For example, Hassan et al. showed that ingested erythrocentaurin can inhibit α-amylase in a concentration-dependent manner, providing a potential treatment for postprandial hyperglycemia [17]. Additionally, erythrocentaurin derivatives were found to have the ability to inhibit the hepatitis B virus [18]. The glucuronic acid conjugate in urine showed better antioxidant capacity than gentiopicrin and could protect against liver cell damage [16]. Based on the above pharmacodynamic and metabolic characteristics of gentiopicrin, it is necessary to further explore the metabolic transformation process of gentiopicrin to understand whether intestinal bacteria metabolize gentiopicrin into other substances and whether this process will affect the absorption of gentiopicrin and pharmacological activities. Finally, we studied whether the metabolites produced had pharmacodynamic or toxic effects. This study is only a preliminary study to further explore gentiopicrin metabolites in intestinal bacteria and to find the material basis of drug efficacy or toxicity in postmortem studies.

The intestinal microecosystem is composed of 10^14^ bacteria from 1100 species [19,20]. The genome encoded by the intestinal microbiome is 150 times more abundant than that of the human genome, providing a rich enzyme library to metabolize drugs [20,21]. Different from liver metabolism, the types of metabolic reactions of intestinal bacteria to drugs include ring cleavage, cyclization, deesterification, dehydrogenation/hydrogenation, demethylation, acetylation, etc. Intestinal bacteria regulate drug metabolism, or their metabolites compete with drugs in metabolic pathways, thus affecting drug metabolism and toxicity [22]. Thus, it is necessary to better understand the pathway by which gentiopicrin is metabolized by the gut microbiota.

In this study, we coincubated intestinal bacteria and liver microsomes with gentiopicrin, respectively. Metabolites were detected by high-performance liquid chromatography coupled with ion trap time-of-flight mass spectrometry (LC/MS^n^-IT-TOF) and an HPLC-MS/MS 8050 system. Gentiopicrin underwent hydrolysis, reduction, and dehydration by intestinal bacteria, and five metabolites were analyzed and identified, including the aglycone reduction products G-M1 and G-M2, the aglycone hydrolysis product G-M3, the erythrocentaurin reduction products G-M4 and G-M6, and the dehydration product G-M5. Three metabolites (G-M3, G-M5, and G-M6) have not been previously reported. This is the first time that the aglycone of gentiopicrin was found to undergo a hydrolysis reaction to produce G-M3, which was then dehydrated by intestinal bacteria to generate G-M5. We also found that G-M1, a precursor of glucuronic acid conjugates found in urine and plasma [16], was produced by intestinal bacteria. However, gentiopicrin was not transformed in liver microsomes. This study provides both new insight regarding the investigation of effective substances and an exploration of the pharmacodynamic and toxicological study of gentiopicrin.

## 2. Results

### 2.1. Biotransformation of Gentiopicrin by Intestinal Bacteria and Liver Microsomes

**Figure 1:**
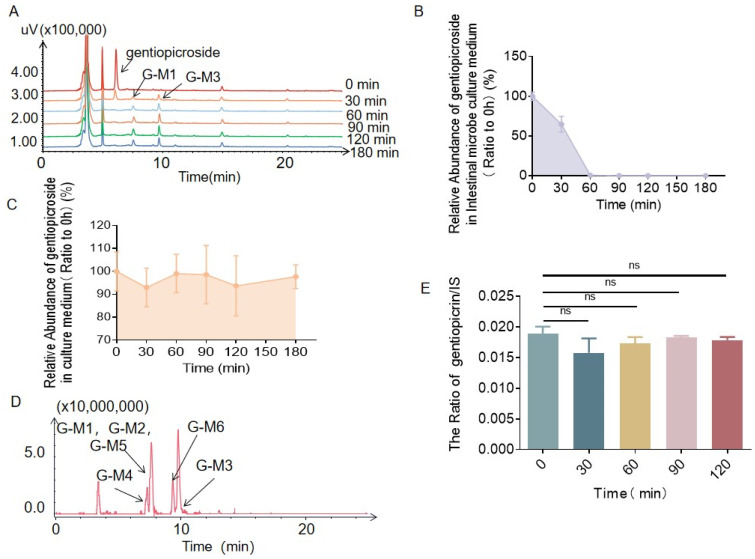
HPLC chromatograms showing that the content of gentiopicrin decreased and the contents of metabolites G-M1 and G-M3 increased with increasing incubation time (**A**). The level of gentiopicrin decreased after incubation with rat intestinal bacteria for 0 min, 30 min, 60 min, 90 min, 120 min, and 180 min (**B**). The level of gentiopicrin changed only slightly in anaerobic medium after 0 min, 30 min, 60 min, 90 min, 120 min, and 180 min of incubation (**C**). Total ion chromatograms (TICs) showing that there were five metabolites in the extract of gentiopicrin incubated with intestinal bacteria (**D**). The level of gentiopicrin changed only slightly in liver microsomes after 0 min, 30 min, 60 min, 90 min, and 120 min of incubation (**E**).

A mixture of anaerobic medium and rat colon contents (containing intestinal bacteria), which were used to investigate the metabolism of gentiopicrin by intestinal bacteria, was prepared as described in Section 4.5. Anaerobic medium was used as a negative control to exclude environmental interference. Gentiopicrin was incubated with rat intestinal bacteria and anaerobic medium for 3 h at 37 °C. According to HPLC analysis (Figure 1A, Figures S1 and S2), the peak area of gentiopicrin decreased with the extension of incubation time, and two main metabolites, G-M1 and G-M3, were present, starting at 30 min. The ratio of the HPLC peak area of gentiopicrin at each time point to the HPLC peak area of gentiopicrin at time point 0 was used to reflect the changes in gentiopicrin with intestinal bacteria in the incubation process. As shown in Figure 1B, gentiopicrin was rapidly metabolized by intestinal bacteria, with 70.23% remaining at 30 min and 0% remaining at 60 min. In the negative control group, after incubation for 3 h at 37 °C, the peak area of gentiopicrin had hardly changed (shown in Figure 1C), suggesting that gentiopicrin can be metabolized by intestinal bacteria. Furthermore, three more metabolites were identified in the total ion chromatogram, namely, G-M4, G-M5, and G-M6 (Figure 1D).

As described in Section 4.6, gentiopicrin was incubated with liver microsomes and the incubation solution was analyzed by LC-MS/MS. By comparing the ratio of the peak area of gentiopicroside and peak area of the internal standard substance at different incubation times, it was found that gentiopicroside was not metabolized in liver microsomes (shown in Figure 1E).

**Figure 2:**
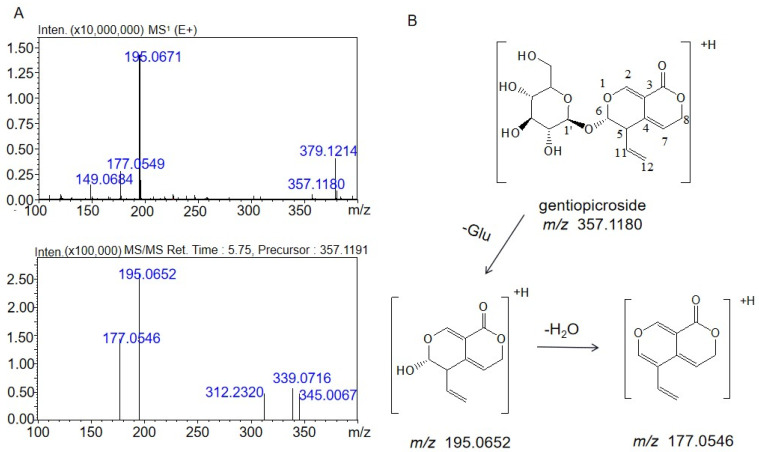
MS^1^ and MS^2^ spectra of gentiopicrin acquired by LC/MS^n^-IT-TOF (**A**). Structures and cleavage pathway of gentiopicrin determined by mass spectrometry (**B**).

In order to analyze the structure of the metabolite, it is necessary to understand the mass spectrum fragmentation characteristics of gentiopicrin. These mass spectral data are shown in Figure 2A. The [M + H]^+^ ion at *m*/*z* 357.1180 was identified as the molecular ion. The fragment at *m*/*z* 195.0652 was formed by cleavage of the glucosidic bond of the parent ion with a loss of 162.0528 Da from the molecular ion ([M-C_6_H_10_O_5_]^+^). The MS^3^ spectrum had a fragment at *m*/*z* 177.0546, which was produced after H_2_O (18.0106 Da) was lost from the fragment at *m*/*z* 195.0652. The structure of gentiopicrin and the cleavage pathway determined by multistage mass spectrometry are shown in Figure 2B.

### 2.2. Identification of the Metabolites Generated by the Intestinal Bacteria

Compared with the control group culture at 0 h, we found six metabolites of gentiopicrin in the intestinal bacterial culture medium. The structures of the metabolites were determined by comparing their accurate molecular masses and fragment ions at each MS^n^ stage with those of the parent drug and literature data. The mass spectral data of each substance are listed in Table 1.

**Figure 3:**
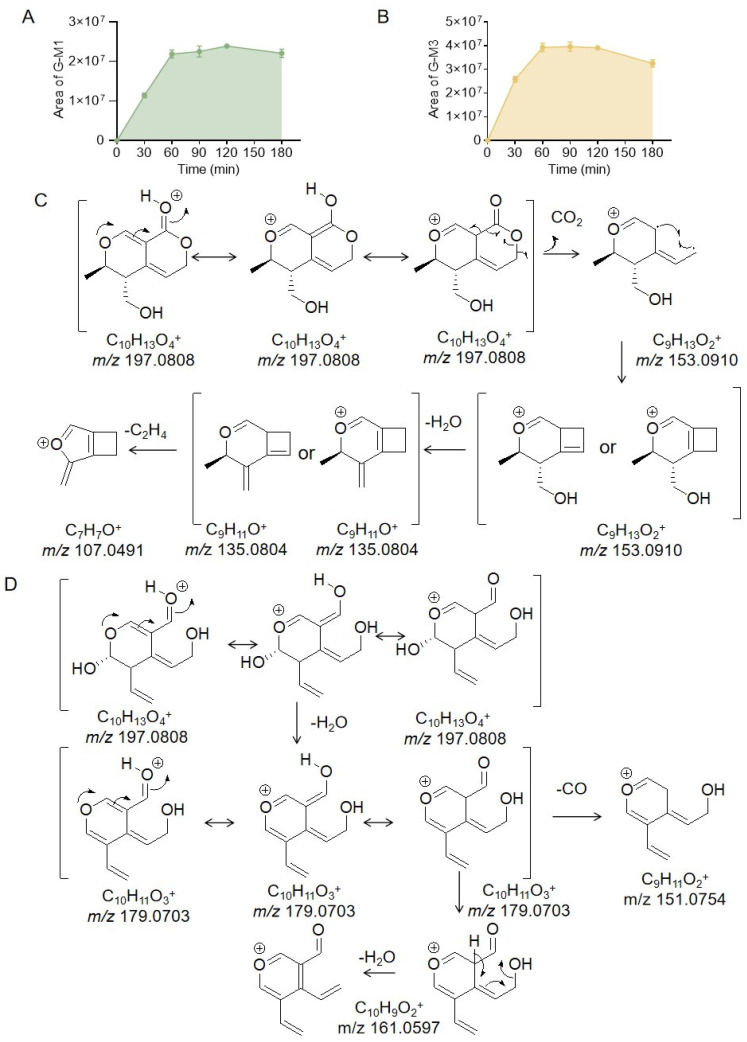
The peak areas of metabolite G-M1 during gentiopicrin incubation with rat intestinal bacteria for 0 min, 30 min, 60 min, 90 min, 120 min, and 180 min (**A**). The peak areas of metabolite G-M3 during gentiopicrin incubation with rat intestinal bacteria for 0 min, 30 min, 60 min, 90 min, 120 min, and 180 min (**B**). Possible structure and cleavage pathway of metabolite G-M1 determined by mass spectrometry (**C**). Possible structure and cleavage pathway of metabolite G-M3 determined by mass spectrometry (**D**).

As shown in Figure 3A,B, the amount of G-M1 increased with the increase in incubation time and reached a plateau at 60–180 min. The peak area of G-M3 reached its maximum at 60 min and then decreased with the increase in time, indicating that G-M3 was further metabolized. G-M1 and G-M3 are isomers with *m*/*z* of 197.0808, which is a loss of 160 Da compared with that of gentiopicrin; thus, these metabolites may be related to gentiopicrin aglycones. However, gentiopicrin aglycones have a molecular weight of 195, which is 2 Da less than those of G-M1 and G-M3, indicating that G-M1 and G-M3 were generated by the further reduction of gentiopicrin aglycones. According to the literature [15], gentiopicrin can form two glucuronic acid conjugates in rat plasma. One of these two metabolites was formed by the direct reduction of the C11-C12 double bond of gentiopicrin aglycone and then binding to glucuronic acid. The other metabolite was generated from the aglycone, where the bond between oxygen-1 and carbon-6 was cleaved, which allowed the formation of an acetal at carbon-6 and the creation of a bond between oxygen-1 and carbon-11. Subsequently, the acetal of carbon-6 was reduced to a hydroxyl and a glucuronic acid moiety was added. The structural characteristics of the precursor compounds of these two binding substances can provide a basis for the identification of the structures of G-M1 and G-M3. The conjugation products of these two glucuronic acids were also reported in another paper [16], and the structures and mass spectral information of these two precursors were also provided. However, the mass spectral data rule only provided the possible structures of G-M1 and G-M3, so NMR analysis was needed to confirm their structures. Then, large amounts of gentiopicrin were incubated with intestinal bacteria in the hope of isolating and preparing the metabolites G-M1 and G-M3 for NMR analysis to confirm their structures. However, only G-M1 was isolated and prepared, and then nuclear magnetic resonance analysis was performed on it. Unfortunately, G-M3 may be unstable; thus, we did not obtain G-M3 for nuclear magnetic resonance analysis. Therefore, G-M3 was identified by comparing the mass spectral data found in the literature with the data obtained in this experiment. The identification analysis process for G-M1 and G-M3 is described as follows.

The secondary fragment ions of G-M1 were 179.0703 and 153.0910. The second-order fragment ion of G-M1 with *m*/*z* 153.0910 was formed by the loss of CO_2_ (43.9898 Da) from the molecular ion, indicating the presence of a lactone structure. Then, the fragment ion at *m*/*z* 153.0910 formed another fragment (*m*/*z* 135.0804) with 18.0106 Da less, indicating the presence of a hydroxyl group. The fragment ion at *m*/*z* 107.0491 was possibly produced by losing 28.0313 Da from the fragment at *m*/*z* 135.0804. However, it is not enough to prove the structure of G-M1 based on the mass spectrum cleavage data. Gentiopicrin (approximately 50 mg) was incubated for 1 h at 37 °C with intestinal bacteria, and G-M1 was then prepared (approximately 4.08 mg, brown powder). By analyzing the NMR carbon and hydrogen data, we found that a single peak in the liquid chromatogram actually consisted of two metabolites (marked as G-M1 and G-M2 in this study), which were chiral isomers. It has been reported that gentiopicrin is transformed into (5*S*,6*S*)-5-(hydroxymethyl)-6-methyl-5,6-dihydro-1*H*,3*H*-pyrano[3,4-*c*]pyran-1-one (**M02**) and (5*R*,6*S*)-5-(hydroxymethyl)-6-methyl-5,6-dihydro-1*H*,3*H*-pyrano[3,4-*c*]pyran-1-one (**M03**) by Penicillium brasiliensis [23], and in our study, the ^1^H-NMR and ^13^C-NMR data for G-M1 and G-M2 were consistent with these two compounds, as shown in Table 2. Therefore, G-M1 and G-M2 were identified as (5S*,6S*)-5-(hydroxymethyl)-6-methyl-5,6-dihydro-1*H*,3*H*-pyrano[3,4-*c*]pyran-1-one and (5R*,6S*)-5-(hydroxymethyl)-6-methyl-5,6-dihydro-1*H*,3*H*-pyrano[3,4-*c*]pyran-1-one, respectively. The NMR spectra are shown in Figures S3–S7. G-M1 and G-M2 had the same cleavage pathways, and that of G-M1 is shown in Figure 3C as an example.

The second-order fragment ion of G-M3 with *m*/*z* 179.0703 was formed from the loss of 18.0105 Da (one H_2_O molecule) from the parent ion at *m*/*z* 197.0808, which indicates that the structure contained hydroxyl groups. Then, the fragment at *m*/*z* 179.0703 lost an additional 18.0106 Da (H_2_O) to generate the fragment ion at *m*/*z* 161.0597 or lost 27.9949 Da to produce the fragment ion at *m*/*z* 151.0754. G-M3 did not show a fragment ion with a loss of 44 Da, but could lose two H_2_O molecules, as described above, demonstrating that G-M3 had two hydroxyl groups but not a lactone structure. However, it is possible that the lactone structure was hydrolyzed to form a hydroxyl and acetal group. There was a fragment with a loss of 27.9949 Da, indicating the presence of a C=O, which clearly indicated that G-M3 might be a product obtained by the direct hydrolysis of the gentiopicrin aglycone lactone ring. This cleavage pathway is shown in Figure 3D. G-M3 was also prepared for nuclear magnetic resonance analysis. After incubating gentiopicrin for an hour, G-M3 could be detected by HPLC. However, G-M3 was hardly detected on the chromatogram when the incubation solution was concentrated, as shown in Figure S8, so G-M3 might be unstable and could not be enriched for preparation.

**Figure 4:**
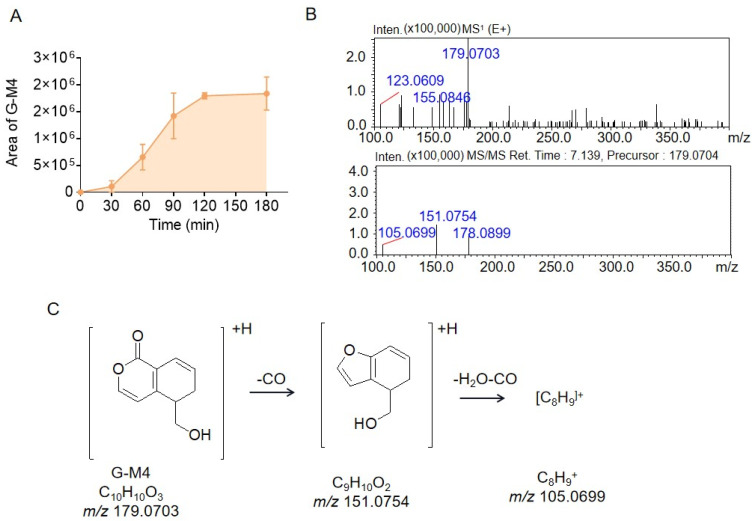
The peak areas of metabolite G-M4 during gentiopicrin incubation with rat intestinal bacteria for 0 min, 30 min, 60 min, 90 min, 120 min, and 180 min (**A**). The MS^1^ and MS^2^ spectra of metabolite G-M4 acquired by LC/MSn-IT-TOF (**B**). Possible structure and cleavage pathway of metabolite G-M4 determined by mass spectrometry (**C**).

M4, G-M5, and G-M6 are isomers with *m*/*z* 179.0703, so they have the same molecular weight of 178. In previous literature [10,11,12], we learned that gentiophora could produce the compound erythrocentaurin under the action of intestinal bacteria, which is a metabolite formed by the isomerization of gentiophorin agricin. According to the molecular weight and structural characteristics of erythrocentaurin, it is helpful for us to analyze the structures of G-M4, G-M5, and G-M6: the molecular weight of erythrocentaurin is 176, which is 2 Da lower than those of G-M4, G-M5, and G-M6, indicating that these three metabolites might be the reduction products of erythrocentaurin. As shown in Figure 4A, the peak area of G-M4 reached a plateau after 2 h of incubation. The fragment ion of G-M4 at *m*/*z* 151.0754 resulted from the loss of 27.9949 Da (one CO group) from the parent ion at *m*/*z* 179.0703. Next, the fragment ion at *m*/*z* 151.0754 lost 46.0005 Da (one CH_2_O_2_ group) to produce the fragment ion at *m*/*z* 105.0699; these mass spectral data are shown in Figure 4B. According to the literature [12], gentiopicrin could be metabolized into 5-(hydroxymethyl)-5,6-dihydroisochromen-1-one by microorganisms, which is consistent with the mass spectral data of G-M4. Therefore, G-M4 was identified as 5-(hydroxymethyl)-5,6-dihydroisochromen-1-one, and the cleavage pathway is shown in Figure 4C.

**Figure 5:**
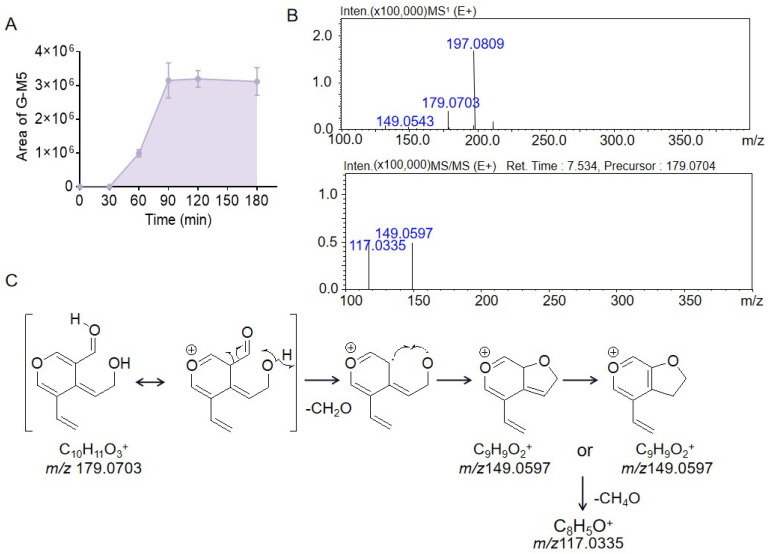
The peak areas of metabolite G-M5 during gentiopicrin incubation with rat intestinal bacteria for 0 min, 30 min, 60 min, 90 min, 120 min, and 180 min (**A**). The MS^1^ and MS^2^ spectra of metabolite G-M5 acquired by LC/MSn-IT-TOF (**B**). Possible structure and cleavage pathway of metabolite G-M5 determined by mass spectrometry (**C**).

Although G-M5 and G-M1 have the same retention time and the same fragment ion *m*/*z* 179.0703, the fragment ions obtained from fragment 179.0703 were different, indicating that they were not the same substance. The peak area of G-M5 gradually increased after incubation for 30 min and reached a plateau at 90 min, as shown in Figure 5A. The fragment ion at *m*/*z* 149.0597 in the MS^2^ spectrum was generated by the loss of 30.0106 Da from the parent ion at m/z 179.0703, suggesting that G-M5 contained a H_2_C=O moiety. However, the spectrum of G-M5 had a fragment ion at *m*/*z* 117.0335, which was 32.0262 Da less than the fragment ion at *m*/*z* 149.0597, which might have been due to the loss of the neutral fragment CH_3_OH. The mass spectrum data are shown in Figure 5B, and the possible structure and cleavage pathway are shown in Figure 5C.

**Figure 6:**
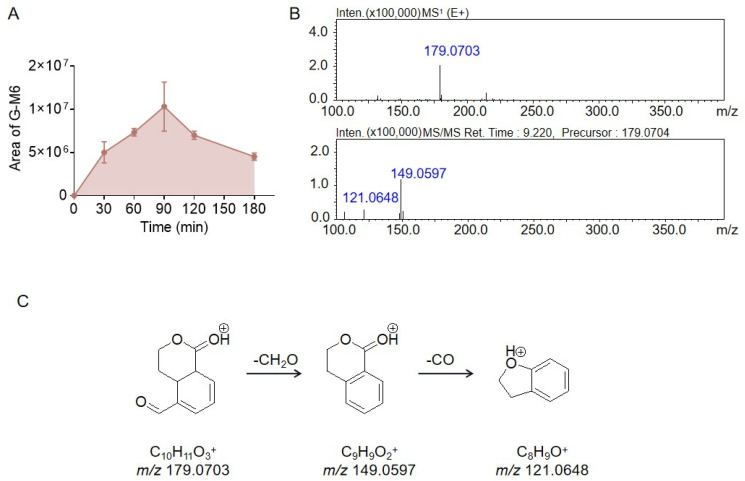
The peak areas of metabolite G-M6 during gentiopicrin incubation with rat intestinal bacteria for 0 min, 30 min, 60 min, 90 min, 120 min, and 180 min (**A**). The MS^1^ and MS^2^ spectra of metabolite G-M6 acquired by LC/MS^n^-IT-TOF (**B**). Possible structure and cleavage pathway of metabolite G-M6 determined by mass spectrometry (**C**).

The peak area of G-M6 peaked at 90 min and then decreased, as shown in Figure 6A. G-M6 had a fragment ion at *m*/*z* 149.0597 in the MS^2^ spectrum, which was produced by the loss of 30.0106 Da from the parent ion at *m*/*z* 179.0703, suggesting that G-M6 also had a H_2_C=O moiety, which also existed in the structure of erythrocentaurin. Third, the G-M6 fragment ion at *m*/*z* 149.0597 could have lost an additional 27.9949 Da to produce the fragment ion at *m*/*z* 121.0648, indicating that the structure of G-M6 had a C=O moiety. Therefore, the structure of G-M6 retained the same lactone and aldehyde groups as erythrocentaurin, and the site of reduction could not be determined only by mass spectrometry data. The mass spectrum data are shown in Figure 6B, and the possible structure and cleavage pathway are shown in Figure 6C.

### 2.3. Proposed Metabolite Pathway of Gentiopicrin

**Figure 7:**
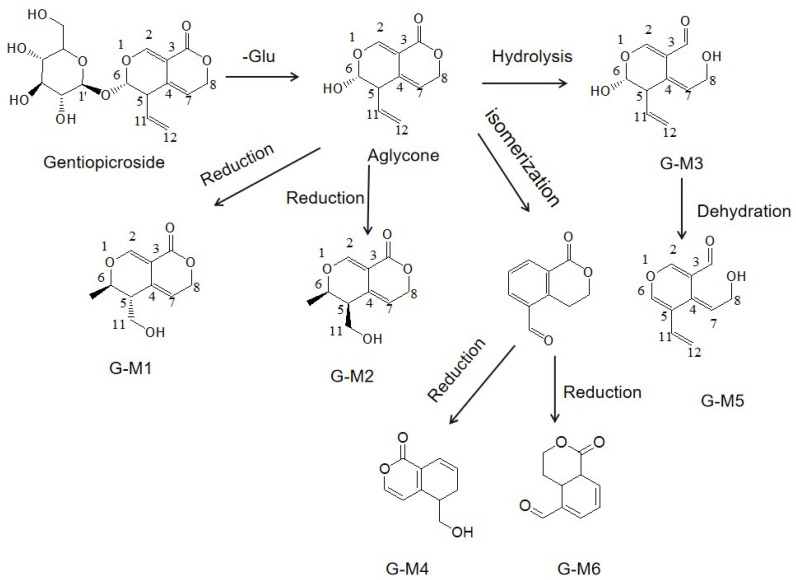
Pathway of gentiopicrin metabolism in intestinal flora in vitro.

In this study, gentiopicrin was incubated in rat intestinal bacteria culture medium, and multistage mass spectral information was obtained by LC/MS^n^-IT-TOF technology. The structures of the metabolites were characterized by multistage mass spectral fragmentation data and literature information to understand the possible route of gentiopicrin metabolism in vitro. In intestinal bacteria, gentiopicrin was generated into the aglycone, where the bond between oxygen-1 and carbon-6 was cleaved, which allowed the formation of an acetal at carbon-6 and the creation of a bond between oxygen-1 and carbon-11. Then, the acetal of carbon-6 was reduced to a hydroxy. In this progress, two metabolites were formed, and they were chiral isomers marked as G-M1 and G-M2. The structures of G-M1 and G-M2 were also confirmed by NMR data, which was the first time that we found chiral metabolites of gentiopicrin. The lactone could be hydrolyzed to form G-M3. As described in the literature [12], aglycones undergo rearrangement; that is, the chemical bond between oxygen-1 and its carbon-6 cleaves, while a bond between C12 and C2 forms. These reactions were followed by the loss of water to form the isocoumarin-type compound erythrocentaurin, which was further reduced to form the final products G-M4 and G-M6. Therefore, we found that gentiopicrin could undergo hydrolysis, reduction, and dehydration by intestinal bacteria. The metabolic pathway of gentiopicrin is summarized in Figure 7.

## 3. Discussion

Intestinal bacteria play an important role in drug metabolism. Intestinal bacteria can produce specific metabolites that are completely different from those produced by liver metabolism, thus affecting the biological benefits of drugs and playing a role in explaining drug efficacy and toxicity [24]. Bacteroides and Firmicutes are dominant in intestinal bacteria, which could encode abundant glycoside hydrolase genes. In addition, intestinal bacteria produce many other types of enzymes, such as reductases and esterases [25,26,27]. Gentiopicrin is in a class of iridoid compounds with both a lactone structure and a glycoside structure. The main metabolites of iridoid glycosides produced by intestinal bacteria are glycosiderin or secondary metabolites of glycosiderin, and the reactions involved include deglycosylation, ester hydrolysis, rearrangement (such as intramolecular cyclization), dehydration, dihydroxylation, dehydrogenation, hydrogenation, and methylation [24]. It was also proven in this study that gentiopicrin could produce the reaction types as reported, and the specific process is shown in Figure 6. The variation in the peak area of each metabolite with incubation time can also indicate the metabolic process of gentiopicrin: G-M1, G-M2, and G-M3 were produced in large quantities after incubation for 30 min, indicating that gentiopicrin was first metabolized to G-M1, G-M2, and G-M3. The peak area of G-M6 began to increase slowly after incubation for 60 min, while the peak area of G-M4 decreased after incubation for 60 min, which was consistent with the hypothesis that G-M6 was a secondary metabolite of G-M4. In the incubation system, we did not detect erythrocentaurin; on the one hand, this may be because erythrocentaurin production was very low and below the detection limit of the instrument. On the other hand, it may be that erythrocentaurin was rapidly metabolized into G-M4 and G-M6, which could be proven by the trend of the peak area of G-M6 and G-M4 with incubation time. Meanwhile, in the coincubation system, gentiopicrin was completely metabolized within one hour, suggesting that gentiopicrin has a faster metabolic rate in intestinal bacteria. It is speculated that the rapid metabolism of gentiopicrin after entering the digestive tract is one of the reasons for its low oral bioavailability.

In this metabolic transformation study, we inferred that the glucuronic acid conjugate of gentiopicrin in vivo may come from G-M1, which is formed by intestinal bacteria, after the formation of the new bond. In addition, 5-(hydroxymethyl)-5,6-dihydroisochromen-1-one [12], which was reported previously, was also observed in our study and named G-M4. G-M4 and G-M6 are coumarin compounds with structures similar to that of erythrocentaurin and may also have pharmacological activity similar to that of erythrocentaurin. Moreover, possible new metabolic pathways of gentiopicrin have been found. For example, G-M4 and G-M6 are generated by the hydrolysis of the aglycone ring and then dehydration or reduction after aglycone isomerization. Both of these compounds have a pyranoid ring, which needs to be further confirmed when studying their efficacy or toxicology. This study provides new clues for the metabolic transformation of gentiopicrin and a material basis for pharmacodynamic and toxicological research.

This study also had certain limitations. For example, there are many reports of the types of metabolic reactions of gentiopicrin or iridoid glycosides and the mass spectrum cleavage of existing metabolites in the literature [9,10,11,12,15,16,25], which provide theoretical support for our analysis of metabolites. The mass spectrum fragmentation information obtained by LC/MS^n^-IT-TOF includes molecular ion, daughter ion, and neutral loss information, which further provides data support for the characterization of metabolites. However, NMR data can provide more evidence for structural analysis. Although G-M1 and G-M2 were obtained by liquid phase preparation, NMR analysis could be used to identify their structures. For the identification of other compounds, due to the stability of compounds or the low yield of metabolites, we did not obtain enough compounds for NMR analysis. Obviously, some metabolites identified in this study cannot be evaluated by preparation and purification for NMR analysis, but we will improve our research through more studies and methods in the future. In addition, the species and abundance of intestinal bacteria may be different between sick and healthy rats, so the metabolites obtained by incubating gentiopicrin with the intestinal bacteria of healthy rats in this study may also be different from those produced under treatment, which requires further exploration.

## 4. Materials and Methods

### 4.1. Instruments and Reagents

Gentiopicrin was purchased from Wuhan Tianzhi Biotechnology Co., Ltd. (Wuhan, China), and its purity was over 98% (HPLC). Isoglycyrrhizin was purchased from Shanghai Standard Technology Co., Ltd. (Shanghai, China), and its purity was over 98% (HPLC). The anaerobic medium was purchased from Beijing Solibao Technology Co., Ltd. (Beijing, China). HPLC-grade methanol and acetonitrile were purchased from Thermo Fisher Scientific (Fair Lawn, NJ, USA), and analytically pure formic acid was purchased from Beijing Sinopharm Holding Chemical Reagents Co., Ltd. (Beijing, China). Pure water was obtained from Hangzhou Wahaha Group Co., Ltd. (Hangzhou, China). Rat liver microsomes were purchased from Beijing InnoChem Science & Technology Co., Ltd. (Beijing, China). The HPLC-MS/MS 8050 system was from Shimadzu Corporation (Kyoto, Japan) and an HPLC–electrospray ionization–ion trap–time-of-flight mass spectrometer (Instrument Model: LCMS-IT-TOF, Shimadzu, Kyoto, Japan) was used to identify the structures of the metabolites of gentiopicrin produced by the intestinal bacteria. An AR-600 MHz (Bruker, Mannheim, Germany) was used to analyze the structure of the compounds. A WH-681 vortex mixer (Scientific Industries, Bohemia, NY, USA) was purchased from Jintan Shenglan Instrument Manufacturing Co., Ltd. (Jintan, China), and a small-scale refrigerated high-speed centrifuge was purchased from Eppendorf (Hamburg, Germany). A METTLER TOLEDO XS105 analytical balance (METTLER, Zurich, Switzerland) was used for weighing compounds.

### 4.2. Animals

SD rats (180–200 g, male) were purchased from Peking University Health Science Center, license number: SCXK (Jing) 2016-0010. All animals had free access to food and water and were housed in a ventilated room with a 12 h light/dark cycle. The temperature was kept at 20–24 °C, while the humidity was kept at 40–60%. Before the trial, the rats were fasted for 12 h and allowed to drink freely. This study was conducted with experimental permission and guidance from the Animal Ethics Committee of the Chinese Academy of Medical Sciences and Peking Union Medical University. All steps referred to the “Organizational Guidelines and Ethics Guidelines of the Experimental Animal Ethics Committee” (Approval number: 00003547).

### 4.3. Identification of Gentiopicrin Metabolites by LC/MS^n^-IT-TOF

Gentiopicrin and its metabolites were separated with an Agilent C8 column (150 mm × 4.6 mm, 5 μm). The mobile phase was composed of eluents A (0.1% formic acid in water, *v*/*v*) and B (acetonitrile), and gradient elution was performed as follows: 20–50% B from 0–7 min, 50–90% B from 7–20 min, and 20% B from 20.01–25 min, with a flow rate of 0.4 mL/min. For IT-TOF analysis, an ESI source was used in the positive mode. Nitrogen was used as the nebulizing gas, and helium was used for MS^n^ analyte fragmentation. The other parameters were as follows: nebulizing gas, 1.5 L/min; CDL temperature, 200 °C; heat block temperature, 200 °C; detector voltage, 1.70 kV; and drying gas pressure, 110 kPa. The CID energy was set at 50%. Mass spectra were acquired in the range of *m*/*z* 100–400 for MS^1^. For the first detection, the MS^n^ data were collected in the automatic mode, and then the parent ion *m*/*z* of 197.0818 and *m*/*z* of 179.0684 were set in the automatic mode to obtain the fragment ion better. The injection volume was 20 µL.

^1^H- and ^13^C-NMR were carried out on an AR-600 MHz (Bruker, Germany) using CD_3_OD as the solvent. The detection parameters were as follows: pulse sequence of zg30, delay time (D1) = 1 s, blank scan times (DS) = 2, and scan times (NS) = 16. All spectra were processed using standard Bruker Topspin software (https://www.bruker.com/en/products-and-solutions/mr/nmr-software/topspin.html, accessed on 8 November 2023).

### 4.4. Determination of Gentiopicrin by LC-MS/MS

The samples of gentiopicrin incubated in liver microsomes were analyzed by an LC-MS/MS 8050 equipped with an ESI ionization source. The analytes were separated through an Agilent C8 column (150 mm × 4.6 mm, 5 μm). The temperature of the column oven was 40 °C and the flow rate was 0.4 mL/min. The mobile phase consisted of formic acid:water (0.1:100, *v*/*v*) (as mobile phase A) and formic acid:acetonitrile (0.1:100, *v*/*v*) (as mobile phase B). The elution gradient conditions (A:B) were as follows: 20–50% B from 0–5 min, 50–90% B from 5–8 min, 90% B from 8–11 min, and 20% B from 11.01–15 min. The detection was carried out in the positive MRM mode, with mass transitions for gentiopicrin of 378.85 → 198.75 (Q1 Pre Bias: −15 V, CE: −20.0 V, Q3 Pre Bias: −11.0 V) and the internal standard (IS, isoglycyrrhizin) of 256.60 → 137.05 (Q1 Pre Bias: −30 V, CE: −23.0 V, Q3 Pre Bias: −21.0 V), respectively. The mass spectrometer parameters were as follows: nebulizer gas, 3.0 L/min; dry gas, 10.0 L/min; heating gas, 10.0 L/min; interface voltage, −4.5 kV; interface temperature, 300 °C; CID gas pressure, 270 kPa; DL temperature, 250 °C; and heat block temperature, 400 °C. The autosampler temperature was maintained at 4 °C.

### 4.5. In Vitro Incubation of Gentiopicrin with Gut Microbiota

The colon contents of SD rats (*n* = 3) were collected and weighed after sacrifice, and then sterilized anaerobic medium (Beijing Solibao Technology Co., Ltd., Beijing, China) was mixed evenly at an *m*/*v* ratio of 1:20 (g/mL). After filtration, the colon contents were cleaned with nitrogen. After mixing thoroughly, bacteria were cultured under anaerobic conditions at 37 °C for 30 min, and then the culture solution of intestinal bacteria was prepared. Gentiopicrin was weighed and dissolved in methanol so that the concentration was 1 mg/mL. An amount of 10 μL of the gentiopicrin solution was added to a presterilized centrifuge tube, followed by the addition of 990 μL of the preincubated enteric bacteria culture medium. In the absence of oxygen, the compound was incubated with the intestinal flora at 37 °C for 0, 30, 60, 90, 120, and 180 min. In addition, gentiopicrin was incubated in anaerobic medium as a negative control for the same lengths of time as noted above. Parallel samples were operated at each time point, and the number was 4. After incubation, twice the volume of methanol was added to the medium to stop the reaction and precipitate the protein. After centrifugation at 12,000 rpm for 10 min, the supernatant was filtered through a membrane (0.45 μm), and then 20 µL was injected into the LC/MS^n^-IT-TOF system for analysis.

### 4.6. In Vitro Incubation of Gentiopicrin with Liver Microsomes

The final microsomes system contained 5 µL of microsomes, 1 µL of gentiopicrin (200 μmol/L), 10 µL of NADPH, and 0.05 mM Tris/HCl (pH = 7.4), which made up a total volume of 200 µL. Then, the systems were incubated for 0, 30, 60, 90, and 120 min. After incubation, 200 µL of cold acetonitrile (containing the internal standard (100 ng/mL)) was added to 100 µL of the incubation solution for protein precipitation. The mixtures were centrifuged at 12,000 rpm for 10 min, and 1 µL of the supernatant was taken for quantitative analysis by LC-MS/MS.

### 4.7. Data Analysis

Data collection and processing were performed using Shimadzu LC-MS Solution (version 5.72, Kyoto, Japan). Statistical analysis was performed using Prism 5 (GraphPad Software, San Diego, CA, USA) with two-tailed analysis of variance and Student’s *t* test. The data are presented as the mean ± standard deviation (SD), and *p* < 0.05 was considered statistically significant.

## Figures and Tables

**Table 1:** Characteristics of the gentiopicroside metabolites in gut microbiota determined by LC/MS^n^-IT-TOF.

Compounds	Reaction	Molecular Formula	Fragment Characteristics		
			MS^1^/[M + H]^+^	MS/MS	MS^3^
Gentiopicrin	-	C_16_H_20_O_9_	357.1180	195.0650	177.0545
G-M1/G-M2	Hydrolysis + Reduction	C_10_H_12_O_4_	197.0808	179.0703 153.0910 135.0804	107.0491
G-M3	Hydrolysis	C_10_H_12_O_4_	197.0808	179.0703	161.0597 151.0754
G-M4	Hydrolysis + Reduction	C_10_H_10_O_3_	179.0703	151.0754	105.0699
G-M5	Hydrolysis + Dehydration	C_10_H_10_O_3_	179.0703	149.0597	117.0335
G-M6	Hydrolysis + Reduction	C_10_H_10_O_3_	179.0703	149.0597	121.0648

**Table 2:** ^1^H-NMR and ^13^C-NMR data for G-M1 and G-M2.

Position	^1^H-NMR(G-M1)	^1^H-NMR (G-M2)	^13^C-NMR (G-M1)	^13^C-NMR (G-M2)
2	7.50 (d, *J* = 1.2)	7.58 (d, *J* = 1.2)	152.9	154.7
3			102.9	103.6
4			126.4	128.9
5	2.45 (ddd, *J* = 7.8; 6.0; 3.0)	2.70 (m)	45.9	44.1
6	4.64 (dq, *J* = 6.6; 3.0)	4.44 (dq, *J* = 6.6; 3.6)	74.9	76.9
7	5.52 (ddd, *J* = 3.6; 3.0; 1.2)	5.53 (ddd, *J* = 3.6; 3.0; 1.2)	115.8	114.3
8	5.07 (dd, *J* = 17.2; 3.0); 4.97 (dd, *J* = 17.2; 3.6)	5.01 (dd, *J* = 11.2; 3.0); 4.97 (dd, *J* = 11.2; 3.6)	70.6	70.6
10			166.8	166.9
11	3.60 (dd, *J* = 14.4; 7.8); 3.52 (dd, *J* = 14.4; 6.0)	3.74 (dd, *J* = 14.4; 6.0); 3.62 (dd, *J* = 14.4; 7.8)	62.5	59.7
12	1.30 (d, *J* = 6.6)	1.36 (d, *J* = 6.6)	18.8	16.4

^1^H-NMR spectra were obtained with CD3OD (600 MHz).

## Data Availability

The data in this study are available in this article.

## Supplementary Materials

The following supporting information can be downloaded at: https://www.mdpi.com/article/10.3390/molecules28227575/s1, Figure S1: HPLC chromatogram of gentiopicrin incubated for 0 min (A). HPLC chromatogram of gentiopicrin incubated for 30 min (B). HPLC chromatogram of gentiopicrin incubated for 30 min (C).; Figure S2: HPLC chromatogram of gentiopicrin incubated for 90 min (A). HPLC chromatogram of gentiopicrin incubated for 120 min (B). HPLC chromatogram of gentiopicrin incubated for 180 min (C).; Figure S3: The ^1^H-NMR spectrum of G-M1 and G-M2.; Figure S4: The ^13^C-NMR spectrum of G-M1 and G-M2.; Figure S5: The ^1^H-^1^H COSY spectrum of G-M1 and G-M2.; Figure S6: The HSQC spectrum of G-M1 and G-M2; Figure S7: The HMBC spectrum of G-M1 and G-M2; Figure S8: HPLC chromatogram of gentiopicrin treated immediately after incubation for 1 h (A). HPLC chromatogram of gentiopicrin incubation solution after concentration (B).
