# Supplementary material for: Metabolic Transformation of Gentiopicrin, a Liver Protective Active Ingredient, Based on Intestinal Bacteria
Source: Molecules. 2023 Nov 14;28(22):7575. doi: 10.3390/molecules28227575 (PMC10673279; doi:10.3390/molecules28227575)
Supplement: Supplementary file 1 [file molecules-28-07575-s001.zip › molecules-2646592-supplementary.pdf]

Metabolic Transformation of Gentiopicrin, a Liver Protective Active Ingredient, Based on Intestinal Bacteria

Jie Fu, Hang Yu, Qinglan Guo, Yanan Wang, Hui Xu, Jinyue Lu, Jiachun Hu and Yan Wang *

State Key Laboratory of Bioactive Substance and Function of Natural Medicines, Institute of Materia Medica, Chinese Academy of Medical Sciences and Peking Union Medical College, Beijing 100050, China; fujie@imm.ac.cn (J.F.); yuhang@imm.ac.cn (H.Y.); guonina@imm.ac.cn (Q.G.); wangyanan@imm.ac.cn (Y.W.); xuhui@imm.ac.cn (H.X.); lujinyue@imm.ac.cn (J.L.); hujiachun@imm.ac.cn (J.H.)

* Correspondence: wangyan@imm.ac.cn; Tel./Fax: +86-10-6316-523

Raw spectra:

Figure captions:

Figure S1 HPLC chromatogram of gentiopicrin incubated for 0 min. (A) HPLC chromatogram of gentiopicrin incubated for 30 min. (B) HPLC chromatogram of gentiopicrin incubated for 30 min. (C).

Figure S2 HPLC chromatogram of gentiopicrin incubated for 90 min. (A) HPLC chromatogram of gentiopicrin incubated for 120 min. (B) HPLC chromatogram of gentiopicrin incubated for 180 min. (C).

Figure S3 The ^{1}H-NMR spectrum of G-M1 and G-M2**.**

Figure S4 The ^{13}C-NMR spectrum of G-M1 and G-M2**.**

Figure S5 The ^{1}H-^{1}H COSY spectrum of G-M1 and G-M2**.**

Figure S6 The HSQC spectrum of G-M1 and G-M2**.**

Figure S7 The HMBC spectrum of G-M1 and G-M2**.**

Figure S8 HPLC chromatogram of gentiopicrin treated immediately after incubation for 1 h. (A). HPLC chromatogram of gentiopicrin incubation solution after concentration. (B).

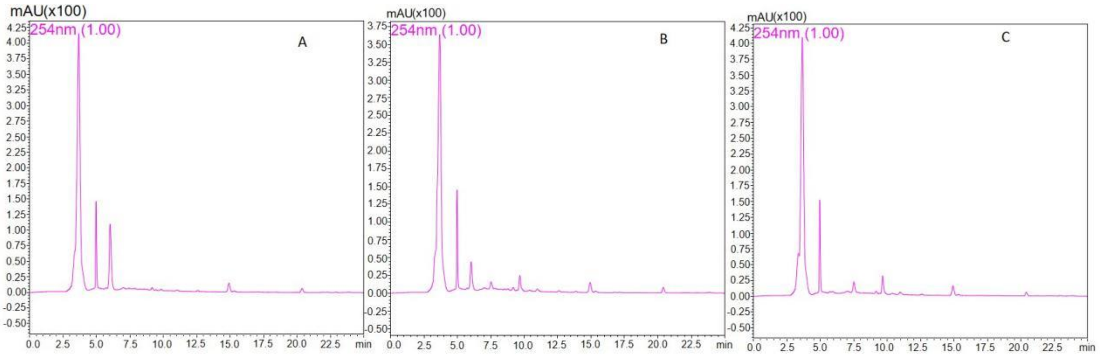

Figure S1 HPLC chromatogram of gentiopicrin incubated for 0 min. (A) HPLC chromatogram of gentiopicrin incubated for 30 min. (B) HPLC chromatogram of gentiopicrin incubated for 30 min. (C).

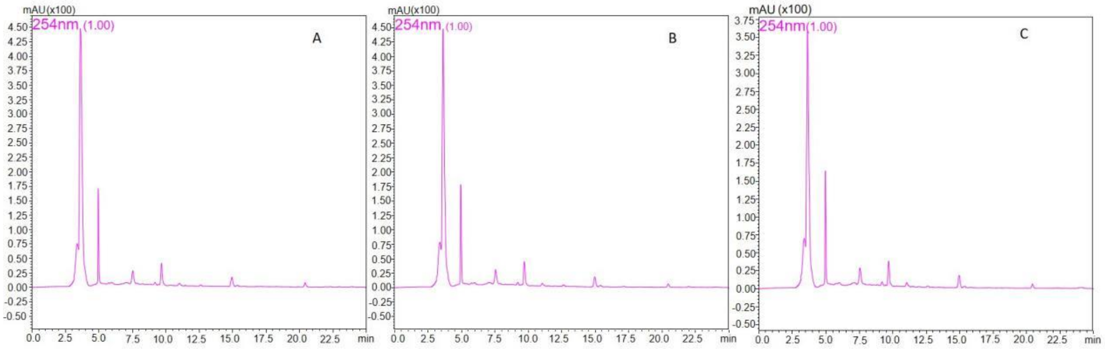

Figure S2 HPLC chromatogram of gentiopicrin incubated for 90 min. (A) HPLC chromatogram of gentiopicrin incubated for 120 min. (B) HPLC chromatogram of gentiopicrin incubated for 180 min. (C).

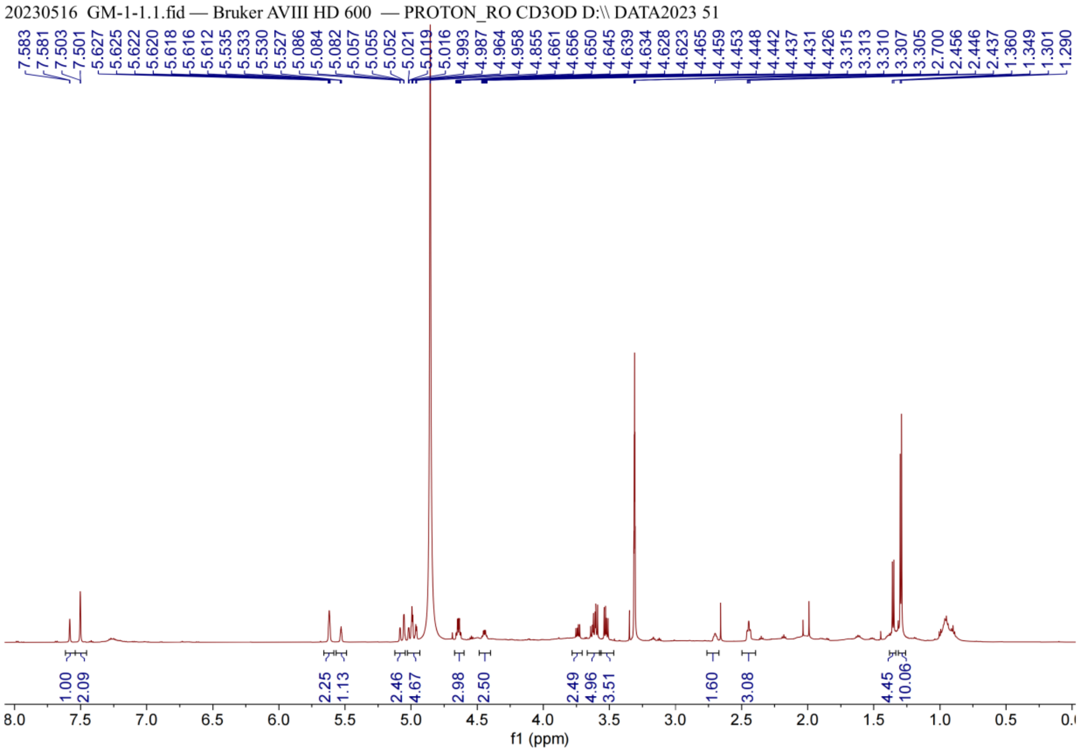

Figure S3 The 1H-NMR spectrum of G-M1 and G-M2.

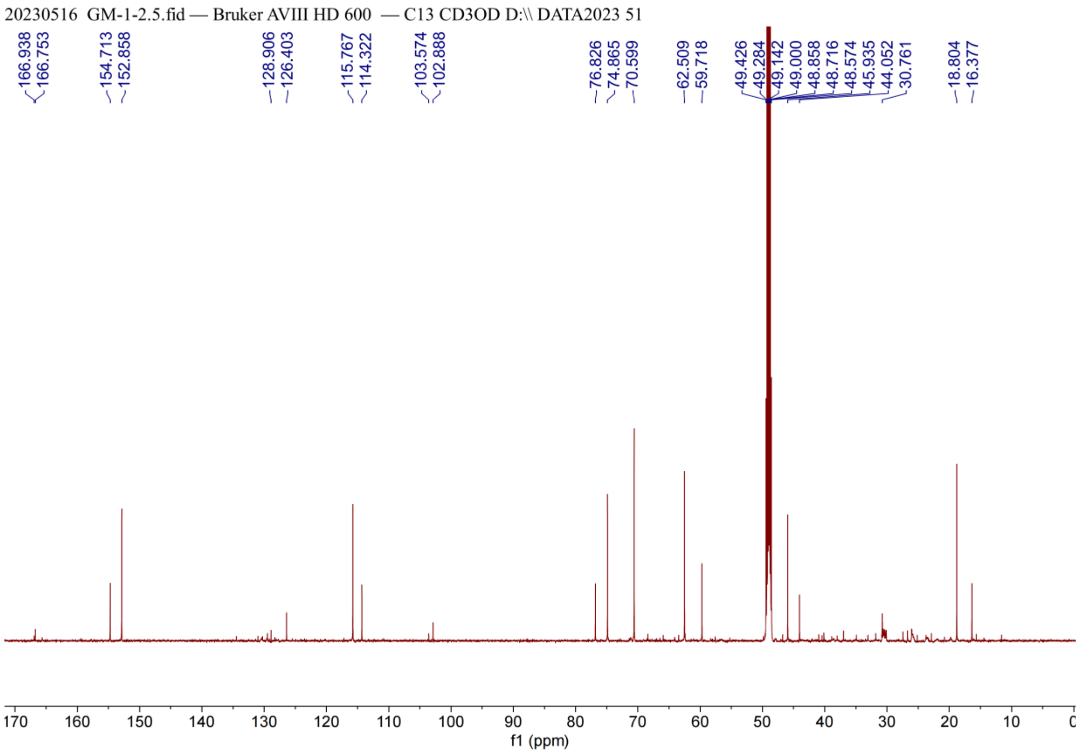

Figure S4 The ^{13}C-NMR spectrum of G-M1 and G-M2**.**

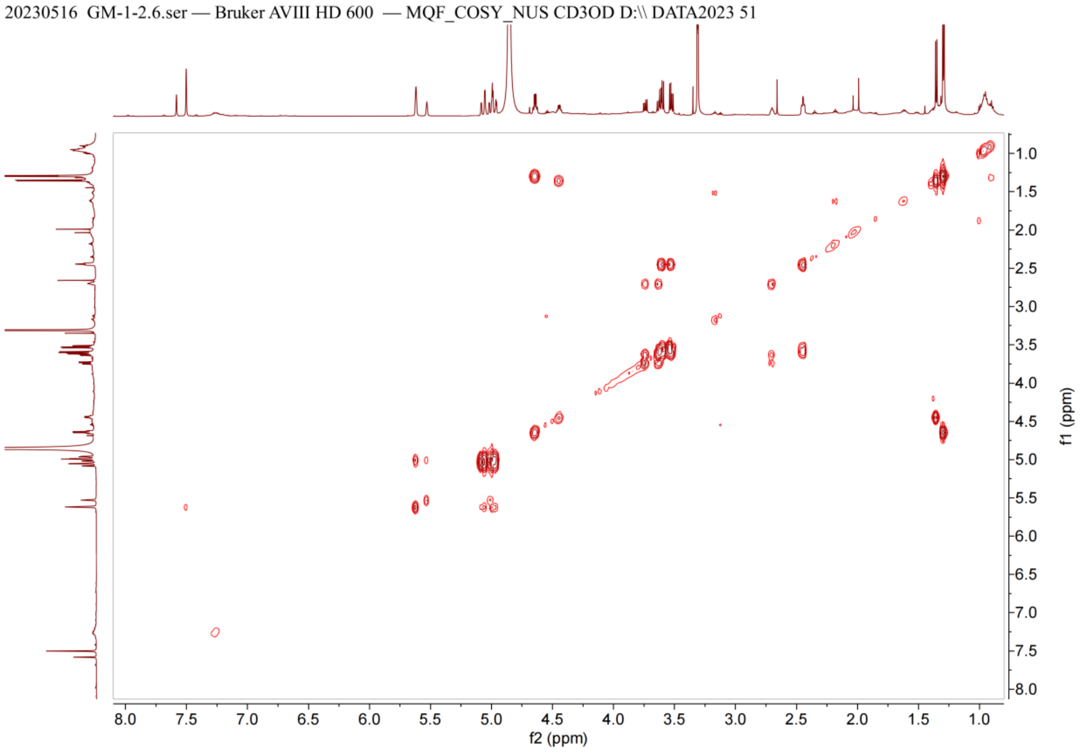

Figure S5 The ^{1}H-^{1}H COSY spectrum of G-M1 and G-M2**.**

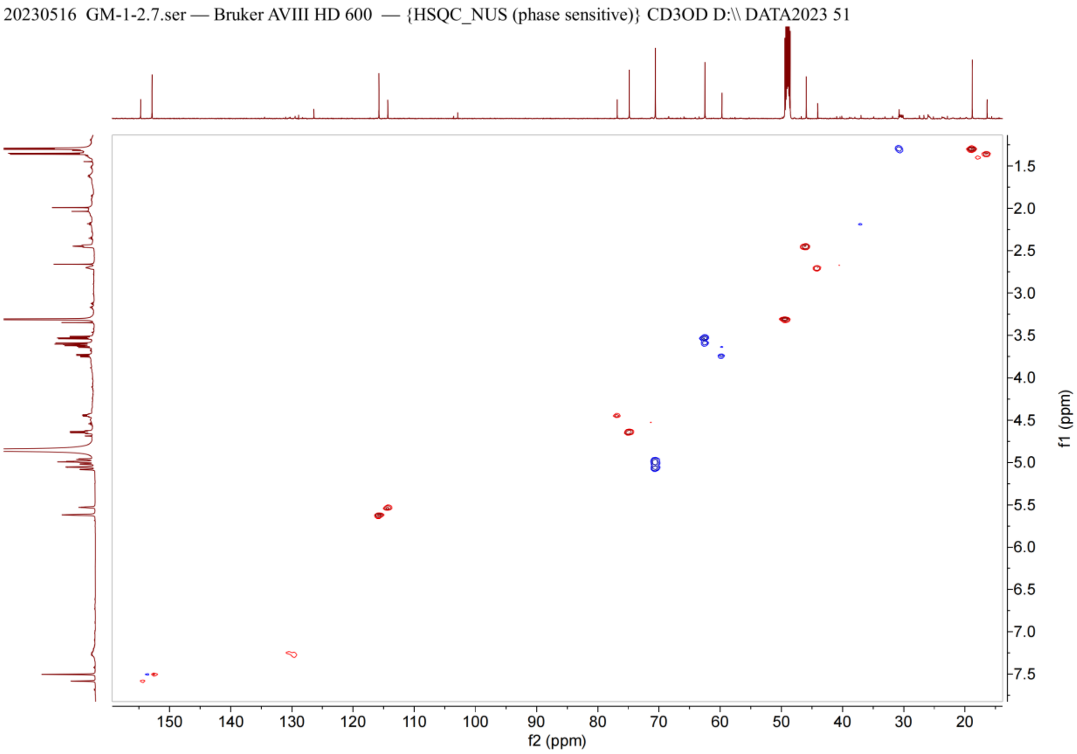

Figure S6 The HSQC spectrum of G-M1 and G-M2.

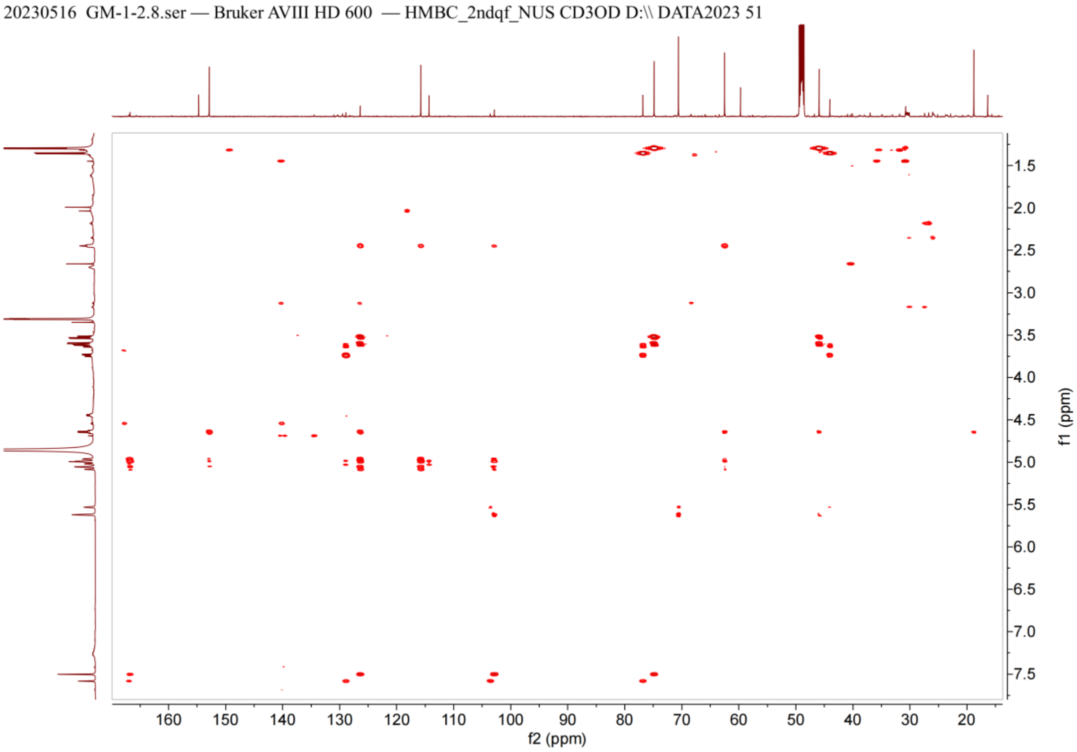

Figure S7 The HMBC spectrum of G-M1 and G-M2.

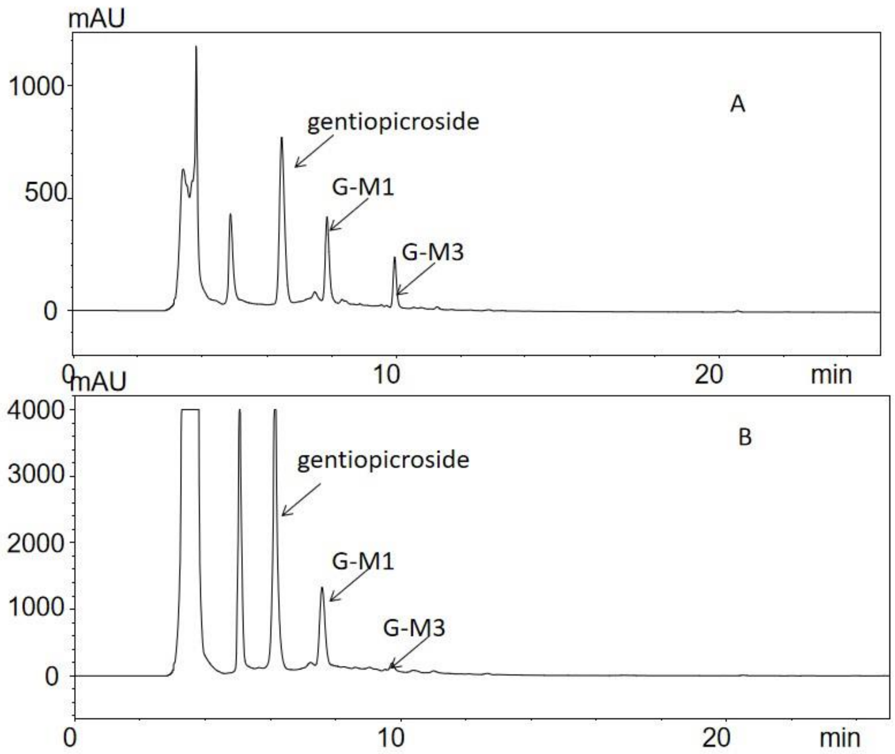

Figure S8 HPLC chromatogram of gentiopicrin treated immediately after incubation for 1 h. (A). HPLC chromatogram of gentiopicrin incubation solution after concentration. (B).
